# Screening of candidate genes for primary open angle glaucoma

**Published:** 2012-07-26

**Authors:** Ting Liu, Lin Xie, Jian Ye, Yuewuyang Liu, Xiangge He

**Affiliations:** 1 Department of Ophthalmology, Daping Hospital, Research Institute of Surgery, Third Military Medical University of PLA, Chongqing, China; 2 Ninth Team of the Cader Brigade of Third Military Medical University of PLA, Chongqing, China

## Abstract

**Purpose:**

Primary open-angle glaucoma (POAG) is one of the leading causes of irreversible blindness in the world. To make progress in understanding POAG, it is necessary to identify more POAG-causing genes.

**Methods:**

Using haplotype analysis, we found that mutational region is located on chromosome 2 in two families. Furthermore, we screened 11 candidate genes on chromosome 2 by protein–protein interaction (PPI) analysis, including mutS homolog 6 (*MSH6*), mutS homolog 2 (*MSH2*), v-rel reticuloendotheliosis viral oncogene homolog (*REL*), endothelial PAS domain protein 1 (*EPAS1*), vaccinia related kinase 2 (*VRK2*), F-box protein 11 (*FBXO11*), EGF containing fibulin-like extracellular matrix protein 1 (*EFEMP1*), reticulon 4 (*RTN4*), RAB1A, member RAS oncogene family (*RAB1A*), ARP2 actin-related protein 2 homolog (*ACTR2*), and calmodulin 2 (phosphorylase kinase, delta; *CALM2*). These 11 genes are all predicted to be related to trabecular meshwork changes and progressive loss of retinal ganglion cells in POAG patients.

**Results:**

According to our study, *FBXO11* and *VRK2* may interact with tumor protein p53 to regulate mitochondrial membrane permeability, mitochondrial membrane organization, and apoptosis. *MSH2* is responsible for repairing DNA mismatches and *RTN4* is for neuronal regeneration. Therefore, they are supposed to play a negative role in cellular process in POAG. *CALM2* may be involved in retinal ganglion cell death and oxidative damage to cell communication.

**Conclusions:**

The results demonstrate that the genes above may be associated with pathogenesis of POAG.

## Introduction

Primary open-angle glaucoma (POAG) is one of the leading causes of irreversible blindness, affecting over 60 million people worldwide [1]. It is characterized by progressive loss of retinal ganglion cells (RGCs) and nerve fibers, raised intraocular pressure (IOP) and other associated factors. Over the past few decades, several studies have demonstrated that genetic factors have substantially contributed to the pathogenesis of POAG. To date, three causative genes have been identified for POAG, namely myocilin (*MYOC*) on chromosome 1q24.3 (*GLC1A*) [2], optineurin (*OPTN*) on chromosome 10p15–14 (*GLC1E*) [3], and WD repeat domain 36 (*WDR36*) on chromosome 5q22.1 (*GLC1G*), which account for less than 10% of POAG cases [4]. In addition, recent studies have shown that more than 20 other genes may contribute to POAG, such as cytochrome P450, family 1, subfamily B, polypeptide 1 (*CYP1B1*) [5,6], apolipoprotein E (*APOE*) [7], optic atrophy 1 (*OPA1*) [8], neurotrophin-4 (*NTF4*) [9], interleukin-1 (*IL1*) [10], opticin (*OPTC*) [11], retinitis pigmentosa GTPase regulator-interacting protein 1 (*RPGRIP1*) [12], tumor protein p53 (*p53*) [13], ADAM metallopeptidase with thrombospondin type 1 motif, 10 (*ADAMTS10*) [14], secreted protein acidic and rich in cysteine (*SPARC*) [15], aquaporin 1 (*AQP1*), and solute carrier family 4, sodium bicarbonate transporter, and member 10 (*SLC4A10*) [4]. However, these genes are still insufficient to explain all POAG cases, it is necessary to identify more candidate genes associated with the pathogenesis of POAG. In this study, through linkage analysis, we found that mutational region is located on chromosome 2. What’s more, 157 known and predicted genes were screened from public data. Ultimately, PPI analysis was performed and showed that 11 candidate genes, including mutS homolog 6 (*MSH6*), mutS homolog 2 (*MSH2*), v-rel reticuloendotheliosis viral oncogene homolog (*REL*), endothelial PAS domain protein 1 (*EPAS1*), vaccinia related kinase 2 (*VRK2*), F-box protein 11 (*FBXO11*), EGF containing fibulin-like extracellular matrix protein 1 (*EFEMP1*), reticulon 4 (*RTN4*), member RAS oncogene family (*RAB1A*), ARP2 actin-related protein 2 homolog (*ACTR2*), and calmodulin 2 (phosphorylase kinase, delta; *CALM2*), are located on chromosome 2. Our data indicated that these hypothesized genes might play certain roles in the etiology of a complex human disease with genetic contributions.

## Methods

### Subjects

**Figure 1:**
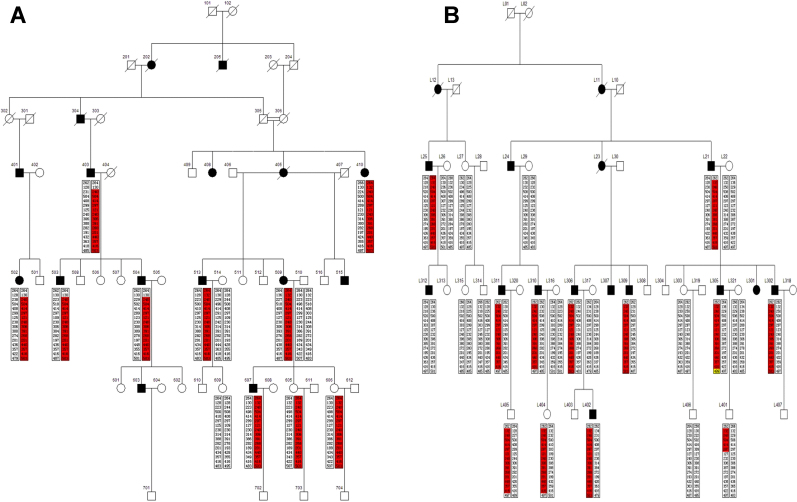
Pedigrees of two Chinese families with primary open-angle glaucoma. Thirteen family members were enrolled in the first family (Family A) and twenty-four members were enrolled in the second family (Family B). The square symbol indicates male; the circle symbol denotes female; the filled symbol represents the affected family member with POAG; and the slashed symbol denotes that the family member is deceased. A phase-known disease haplotype in each family is indicated by a red box. The yellow box is the mutation site.

This project was approved by the Institutional Review Board of Daping Hospital of the Third Military Medical University, PLA. Study subjects consisted of two large Chinese families. Thirteen family members were enrolled in the first family (Family A; Figure 1A) and twenty-four members were enrolled in the second family (Family B; Figure 1B). All family members underwent ophthalmic examination including visual acuity testing, tonometry, gonioscopy, and visual field testing. Clinical diagnosis was based on IOP, vision field loss, angle appearance, and optical disc appearance by the ophthalmologist specializing in glaucoma from Daping Hospital of the Third Military Medical University. The patients were diagnosed with POAG according to the following four criteria: 1) optic damage (cup/disc ratio >0.5); 2) visual field loss found by a Humphrey perimetry test; 3) open-angle appearance by gonioscopy, and 4) an IOP equal to or higher than 22 mmHg while not on medication [16].

### Genotyping and linkage analysis

A genome wide linkage scan was performed in both Family A and B using ABI Linkage Mapping Set v2.5, which contained 411 short tandem repeat (STR) markers and True Allele PCR Premix (ABI, Foster City, CA) according to the manufacturer’s instructions. The amplified polymerase chain reaction (PCR) products were loaded on to the ABI 3100 Genetic Analyzer (ABI). We used the MLINK of the LINKAGE (v.5.1; Cambridge, UK) program to calculate two-point logarithm of odds (LOD) scores [17]. An autosomal dominant mode of inheritance with full penetrance and a disease allele frequency of 0.01 were assumed in the calculations. For fine mapping, additional STR markers were chosen from the Marshfield database.

### POAG disease genes collection

The Indian Genetic Disease Database (IGDD) [18] integrated and curated repository of growing number of mutation data on common genetic diseases afflicting the Indian populations. The database covers 52 diseases with information on 5760 individuals carrying the mutant alleles of causal genes. Finally, *CYP1B1, MYOC, OPTN*, and *OPTC* were selected. The GeneCards [19] is a searchable and integrated database of human genes that provides concise genomic related information on all known and predicted human genes. A total of 153 POAG-related genes were collected. Online Mendelian Inheritance in Man (OMIM) [20] is a continuously updated catalog of human genes, genetic disorders and traits, which particularly focuses on the molecular relationship between genetic variation and phenotypic expression. It is thus considered to be a phenotypic companion to the Human Genome Project. After searching the Phenotype OMIM number 137760, three POAG related genes, *CYP1B1, GLC1B* and *OPTN* were collected. GENATLAS was also used to screen the potential POAG related genes, and *GLC1E* and *GLC1L* were obtained. At last, 157 unique POAG disease genes were collected from the above databases after removing the repetitive genes.

### PPI and regulation network construction

The Human Protein Reference Database (HPRD) [21] is a protein database accessible on the internet. The Biologic General Repository for Interaction Data sets (BioGRID) [22] is a curated biologic database of protein–protein and genetic interactions. PPI data were obtained from the HPRD and BioGRID database. A total of 326119 unique human PPI pairs were collected, among which 39240 pairs were from HPRD and 379426 pairs were from BioGRID. The total number of unique PPI pairs is less than the sum of the two sets due to overlap and duplication.

The TRANSFAC database contains data on transcription factors, including their experimentally-proven binding sites and regulated genes [23]. The Transcriptional Regulatory Element Database (TRED) has been built in response to increasing needs of an integrated repository for both cis- and trans- regulatory elements in mammals [24]. TRED made the curation for transcriptional regulation information, including transcription factor binding motifs and experimental evidence. The curation is currently focused on target genes of 36 cancer-related TF families. Seven hundred seventy-four pairs of regulatory relationship between 219 transcription factors (TFs) and 265 target genes were collected from TRANSFAC. Five thousand seven hundred twenty-two pairs of regulatory relationship between 102 transcription factors (TFs) and 2,920 target genes were collected from TRED. A total of 6,328 pairs of regulatory relationships between 276 TFs and 3,002 target genes were collected by combination of the two regulation data sets.

Using the PPI data that collected from HPRD and BioGRID, and regulation data that collected from TRANSFAC and TRED, we mapped the POAG disease genes to target genes on chromosome 2p ranged from 46411503 to 65629132.

### GO enrichment analysis

The Gene-Ontology database (GO) provides a useful tool to annotate and analyze the function of large numbers of genes. To learn about the biology in certain gene sets, it is desirable to find functional annotation or Gene-Ontology groups which are highly represented in the gene sets. The program (GOstat) facilitates the analysis of such gene sets, provides statistics about the GO terms contained in the data and sorts the GO annotations giving the most representative GO terms first [25]. We used the default parameters with the count >3 and Benjamini corrected p-value <0.01 to search overrepresented GO terms in Biologic Process.

## Results

### Linkage analysis

The linkage analysis results showed that the two families shared the same disease haplotype, suggesting they inherited the same mutation from a common founder. The disease interval was defined to 14.11 cM between D2S391 (70.31 cM) and D2S2231 (84.42 cM) using refining STR markers and haplotype analysis. The LOD scores in these two Chinese families were listed in Table 1.

**Table 1:** Two-point LOD scores between short tandem repeats and disease phenotype.

** **	** **	**Two-point LOD score of Family A and B (θ)**							** **
**STR marker**	**Genetic position (cM)**	**0.00**	**0.01**	**0.05**	**0.10**	**0.20**	**0.30**	**0.40**	**Max LOD score (θ)**
D2S2298	65.94	-3.10	-1.55	-0.49	-0.13	0.06	0.05	0.01	0.07 (0.24)
D2S391	70.31	-12.55	-7.66	-4.08	-2.45	-1.08	-0.55	-0.29	-0.03 (0.49)
D2S2739	73.61	-7.48	-4.65	-1.96	-0.84	-0.11	0.01	-0.04	0.01 (0.29)
D2S123	73.61	-8.97	-6.18	-3.39	-2.12	-1.05	-0.56	-0.26	-0.02 (0.49)
D2S2369	73.61	-3.67	-2.78	-1.46	-0.86	-0.37	-0.19	-0.08	-0.01 (0.49)
D2S2352	76.34	-4.50	-2.68	-1.27	-0.71	-0.33	-0.21	-0.13	-0.02 (0.49)
D2S2153	76.88	-4.11	-1.47	0.25	0.81	0.89	0.56	0.18	0.94 (0.16)
D2S378	77.43	-4.33	-2.37	-0.87	-0.278	0.09	0.12	0.06	0.13 (0.27)
D2S2279	77.97	1.04	1.00	0.82	0.62	0.31	0.11	0.01	1.04 (0.00)
D2S393	80.16	-3.67	-1.80	-0.40	0.10	0.33	0.27	0.13	0.33 (0.21)
D2S357	80.16	-4.28	-2.04	-0.32	0.26	0.45	0.29	0.07	0.46 (0.18)
D2S2332	80.69	-0.34	1.57	1.93	1.83	1.83	0.81	0.33	1.93 (0.05)
D2S2206	81.49	-5.26	-2.75	-0.58	0.24	0.59	0.44	0.17	0.59 (0.20)
D2S2320	82.29	-3.56	-1.85	-0.53	-0.09	0.05	-0.06	-0.13	0.05 (0.18)
D2S2397	82.82	0.60	0.59	0.55	0.49	0.36	0.22	0.10	0.60 (0.00)
D2S2231	84.42	-5.76	-4.20	-1.71	-0.58	0.14	0.22	0.12	0.22 (0.27)
** **	** **	**Two-point LOD score of Family A (θ)**							** **
**STR marker**	**Genetic position (cM)**	**0.00**	**0.01**	**0.05**	**0.10**	**0.20**	**0.30**	**0.40**	**LOD_max_ score (θ)**
D2S2298	65.94	0.15	0.14	0.10	0.06	0.01	0.00	0.000	0.15 (0.00)
D2S391	70.31	-5.70	-3.37	-1.77	-1.03	-0.41	-0.15	-0.04	-0.00 (0.49)
D2S2739	73.61	-1.74	-1.17	-0.38	-0.08	0.07	0.06	0.03	0.07 (0.23)
D2S123	73.61	-2.27	-1.70	-0.87	-0.51	-0.23	-0.11	-0.03	-0.00 (0.49)
D2S2369	73.61	-2.44	-1.84	-0.94	-0.53	-0.19	-0.06	-0.01	0.00 (0.49)
D2S2352	76.34	0.57	0.55	0.46	0.36	0.20	0.08	0.02	0.57 (0.00)
D2S2153	76.88	-1.76	-1.20	-0.39	-0.08	0.08	0.07	0.03	0.09 (0.23)
D2S378	77.43	-2.68	-2.03	-1.08	-0.63	-0.24	-0.09	-0.02	0.00 (0.49)
D2S2279	77.97	0.20	0.18	0.10	0.02	-0.06	-0.06	-0.03	0.20 (0.00)
D2S393	80.16	-2.30	-1.71	-0.85	-0.46	-0.16	-0.04	-0.00	0.00 (0.46)
D2S357	80.16	-2.37	-1.80	-0.98	-0.62	-0.33	-0.17	-0.05	-0.00 (0.49)
D2S2332	80.69	0.88	0.85	0.73	0.59	0.35	0.17	0.06	0.88 (0.00)
D2S2206	81.49	-1.78	-1.22	-0.42	-0.11	0.05	0.05	0.03	0.05 (0.25)
D2S2320	84.29	1.06	1.03	0.89	0.72	0.43	0.21	0.07	1.06 (0.00)
D2S2397	82.82	0.23	0.23	0.22	0.19	0.13	0.07	0.02	0.23 (0.00)
D2S2231	84.42	-1.37	-0.81	-0.06	0.20	0.26	0.16	0.07	0.27 (0.16)
** **	** **	**Two-point LOD score of Family B (θ)**							** **
**STR marker**	**Genetic position (cM)**	**0.00**	**0.01**	**0.05**	**0.10**	**0.20**	**0.30**	**0.40**	**LOD_max_ score (θ)**
D2S2298	65.94	-3.25	-1.69	-0.60	-0.19	0.04	0.05	0.01	0.06 (0.25)
D2S391	70.31	-6.85	-4.29	-2.31	-1.41	-0.67	-0.40	-0.25	-0.03 (0.49)
D2S2739	73.61	-5.74	-3.48	-1.58	-0.76	-0.18	-0.05	-0.07	-0.02 (0.49)
D2S123	73.61	-6.70	-4.48	-2.52	-1.61	-0.82	-0.45	-0.22	-0.02 (0.49)
D2S2369	73.61	-1.23	-0.94	-0.52	-0.33	-0.19	-0.13	-0.07	-0.01 (0.49)
D2S2352	76.34	-5.08	-3.25	-1.75	-1.08	-0.54	-0.29	-0.16	-0.02 (0.49)
D2S2153	76.88	-2.35	-0.28	0.64	0.89	0.81	0.49	0.15	0.91 (0.13)
D2S378	77.43	-1.65	-0.34	0.21	0.35	0.33	0.20	0.08	0.37 (0.13)
D2S2279	77.97	0.84	0.82	0.72	0.60	0.37	0.17	0.04	0.84 (0.00)
D2S393	80.16	-1.36	-0.09	0.45	0.56	0.49	0.32	0.14	0.56 (0.12)
D2S357	80.16	-1.91	-0.25	0.65	0.88	0.78	0.46	0.13	0.90 (0.12)
D2S2332	80.69	-1.22	0.72	1.20	1.24	1.00	0.64	0.27	1.25 (0.08)
D2S2206	81.49	-3.47	-1.54	-0.15	0.35	0.54	0.39	0.14	0.55 (0.19)
D2S2320	84.29	-4.62	-2.87	-1.42	-0.82	-0.39	-0.27	-0.20	-0.03 (0.49)
D2S2397	82.82	0.37	0.36	0.34	0.30	0.23	0.16	0.08	0.37 (0.00)
D2S2231	84.42	-4.40	-3.39	-1.66	-0.78	-0.12	0.05	0.05	0.06 (0.34)

### PPI and regulation network construction

**Figure 2:**
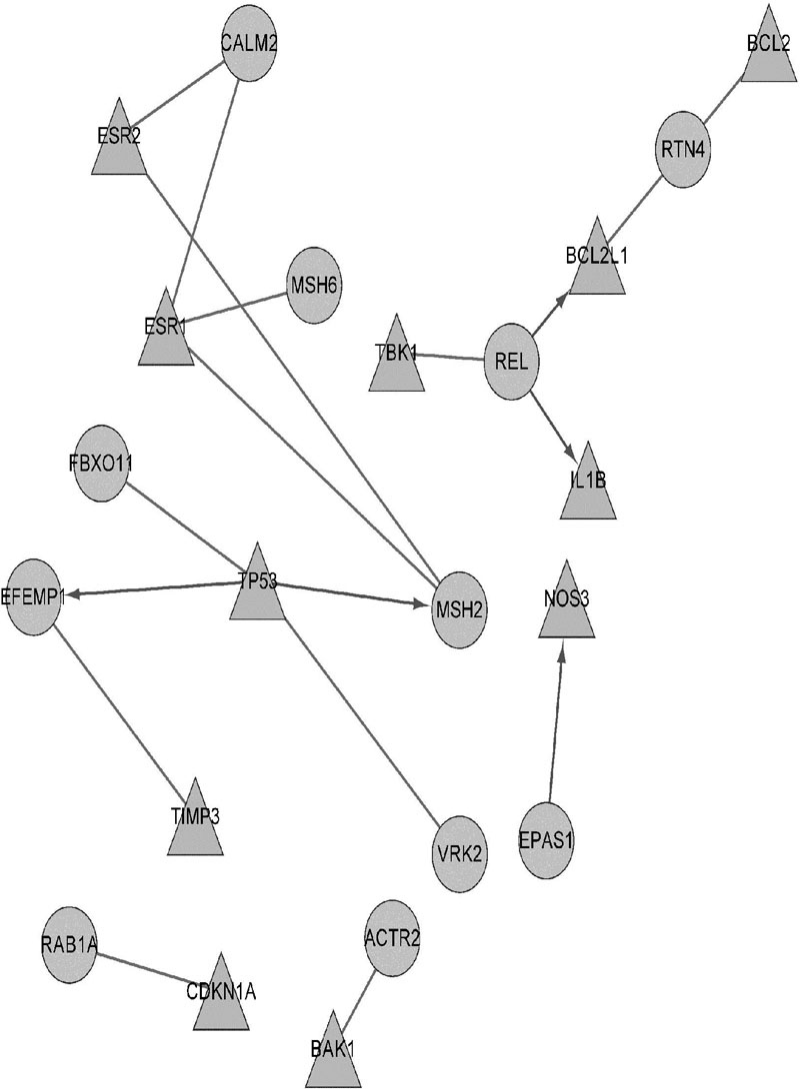
Network between targets and POAG disease genes. Directed edge means the source nodes regulated the target nodes; the undirected edges mean the PPI relationships. The triangle nodes stand for POAG genes proved by the previous analysis on the database. The ellipse stands for target genes on chromosome 2: 46411503–65629132.

After searching the genes in the range from 46411503 to 65629132 of chromosome 2 (NCBI homo reference, BUILD 37.2), 168 target genes were detected, among which 90 targets could be mapped to the gene symbol. Furthermore, a total of 157 unique POAG disease associated genes were obtained from the IGDD, OMIM, GENATLAS, and GeneCards databases. The PPI and regulation data sets were employed to build the network between 90 targets and 157 POAG disease genes. Figure 2 shows the relationship between 11 targets and the 11 POAG related genes. Finally, these 11 targets of *MSH6, REL, MSH2, EPAS1, VRK2, FBXO11, EFEMP1, RTN4, RAB1A, ACTR2*, and *CALM2* were located on chromosome 2: 46411503–65629132, which have been shown to be closely related to POAG.

To assess the significance of the network, we used the over-represented GO terms of GOstats [25]. Enrichment analysis by the hypergeometric distribution was used to find the significant GO terms and pathways. Some of the GO terms were enriched among these genes in the network, including regulation of mitochondrial membrane permeability, mitochondrial membrane organization and negative regulation of cellular process, and others (Table 2).

**Table 2:** GO enrichment analysis.

GO** terms**	**Des**	**count**	**FDR**
GO:0046902	regulation of mitochondrial membrane permeability	4	3.20E-10
GO:0007006	mitochondrial membrane organization	4	1.23E-07
GO:0048523	negative regulation of cellular process	10	1.23E-07
GO:0048519	negative regulation of biologic process	10	1.34E-07
GO:0006839	mitochondrial transport	4	2.34E-06
GO:0006915	apoptosis	8	2.34E-06
GO:0051881	regulation of mitochondrial membrane potential	3	2.34E-06
GO:0012501	programmed cell death	8	2.34E-06
GO:0042981	regulation of apoptosis	7	2.34E-06
GO:0043067	regulation of programmed cell death	7	2.34E-06
GO:0016265	death	8	2.34E-06
GO:0008219	cell death	8	2.34E-06
GO:0001836	release of cytochrome c from mitochondria	3	2.50E-06
GO:0048522	positive regulation of cellular process	8	2.75E-06
GO:0007154	cell communication	15	2.75E-06

FDR: False Discovery Rate (Benjamini).

## Discussion

POAG is characterized by specific morphologic and biochemical changes in trabecular meshwork (TM), including loss of TM cells, accumulation of extracellular matrix (ECM), and accelerated senescence. ECM deposition in the juxtacanalicular region of TM involves increased expression of transforming growth factor (TGF)-β2 in the aqueous humor, but this effect is blunted or prevented if bone morphogenetic protein 7 (BMP7) is added. Further study indicates that the expression level of *EFEMP1* can be upregulated by TGF-β2 and down-regulated by BMP7 in POAG progression [26]. Mutations in *EFEMP1* result in macular dystrophy with subretinal deposits [27,28].

To date, several factors have been proposed to influence the RGCs loss in POAG patients, such as mechanical stress due to increased IOP, reperfusion injury, oxidative stress, glutamate excitotoxicity, and autoimmunity [29]. Abnormally elevated IOP elicits a complex sequence of putative neurodestructive cellular responses in the optic nerve head [30]. A prominent astrocyte reactivation serves as the basis for these responses [31], in which four nuclear factor of kappa light polypeptide gene enhancer in B-cells (NF-κB) subunits, v-rel reticuloendotheliosis viral oncogene homolog (avian; c-REL), v-rel reticuloendotheliosis viral oncogene homolog A (avian; RELA), nuclear factor of kappa light polypeptide gene enhancer in B-cells 2 (p49/p100) (NFKB2), and nuclear factor of kappa light polypeptide gene enhancer in B-cells inhibitor, alpha (NFKBIA), are activated at transcriptional level [32]. In addition, there is a significant increase in transcription of Golgi-resident protein transcripts, including *RAB1A*. Remodeling or redistribution of actin at cellular edges is an essential part of establishing cell polarity and the formation of processes in astrocytes. Actin polymerization may be regulated by ARP protein complex (*ACTR2*, *WSP*) [33].

*FBXO11* is a member of the F-box protein sub-family and a component of the Skp1 Cullin1 F-box (SCF) complex. Some studies have presented that *FBXO11* promotes the neddylation of p53 both on two lysines, Lys-320 and Lys-321 to suppress p53 function [34]. This is consistent with recent studies showing that retinal cells from mice carrying a homozygous lysine 317 to arginine mutation, the site corresponding to human lysine 320, undergo increased p53-dependent apoptosis in response to DNA damage [35]. These data suggest that *FBXO11* is important for p53 repression by neddylation at lysine 320, and reduced expression of p53 shows resistance to RGCs death of POAG [36]. However, some studies indicate that *VRK2* has an identical catalytic NH_2_-terminal domain and could phosphorylate p53 in vitro uniquely at Thr18 [37]. Phosphorylation of p53 protein may lead to RGCs death [38]. Therefore, *FBXO11* and *VRK2* may have an antagonistic effect to regulate p53 expression and indirectly influence POAG. Analysis of GO terms demonstrates that p53 is involved in the regulation of mitochondrial membrane permeability, mitochondrial membrane organization, negative regulation of cellular process, and apoptosis.

The increased expression and activity of inducible nitric oxide synthase (iNOS) in the TM of patients with POAG accord with the visual field defect. However, neuronal and endothelial NOS are constitutive, with Ca^2+^/calmodulin-dependent enzymes tightly controlled by mechanisms regulating physiologic intracellular Ca^2+^ levels [39]. Calcium-binding proteins are frequently overexpressed in RGCs, including calmodulin-1, calmodulin-2, calmodulin-3, calretinin, calpactin 1 and calcyclin [40]. These data indicated that calmodulin 2 is associated with cell communication (GO term analysis).

Oxidative DNA damage is closely associated with IOP (maximum values and fluctuation) and visual field defects, as detected by computerized ocular field analysis [41], which may induce upregulation of various DNA repair genes, such as *MSH6* and *MSH2*. MSH2-MSH6 protein can bind to the mismatch site through a conserved motif of domain I of chain MSH6, whereas the clamps (domain IV) interact with the DNA backbone to stabilize a highly bent DNA structure [42]. This is consistent with our GO term analysis that MSH2 plays a negative regulation in cellular process.

It is also known that retinal ischemia is a major cause of visual impairment and glaucoma development. Mild retinal ischemia after 3 h of reperfusion might exclusively induce reticulon family member reticulon (*Nogo/Rtn4*) gene participating in neuronal regeneration [43]. The EyeSAGE database analysis has also indicated that *RTN4* is a possible prioritized gene for POAG [44]. These views accord with our GO terms analysis results, which revealed that *RTN4* plays a negative regulatory role in cellular process. It is worthy of note that, hypoxia plays a key role in ischemic and neovascular disorders of the retina. Cellular responses to oxygen are mediated by hypoxia-inducible transcription factors (HIFs), such as HIF-2alpha (also known as EPAS1), which has been found overexpressed in Müller glia and astrocytes by post-translational stabilization [45].

### Conclusions

Eleven candidate genes for POAG are located on chromosome 2 according to the PPI analysis. It is possible that these genes may play different roles in the cellular activity. For example, *MSH2* and *MSH6* are responsible for repairing DNA mismatch. *ACTR2* regulates actin distribution and establishes cell polarity in astrocytes. Moreover, the candidate genes might work together to regulate cell activity, as evidenced by the antagonistic role of *FBXO11* and *VRK2* in regulating apoptosis of RGCs. Together, the 11 candidate genes may participate in TM changes and progressive loss of RGCs in POAG patients. Our study provided some clues as to the further functional analysis of the candidate genes. It will be of great importance to investigate in the future how one gene or several genes act their roles coordinately in response to the activation of certain cell signaling pathway. Elucidation of these questions will undoubtedly help understand the molecular mechanisms of the POAG pathogenesis.
